# Older Women Causing a Ruckus: Gentrification, Displacement, and Tenant Advocacy

**DOI:** 10.1093/geroni/igaa057.2495

**Published:** 2020-12-16

**Authors:** H Shellae Versey

**Affiliations:** Wesleyan University, Middletown, Connecticut, United States

## Abstract

Gentrification is a process through which lower-income neighborhoods experience large-scale investments and an influx of wealthier residents, often displacing lower-income residents. The restructuring of neighborhoods for newer, wealthier residents can compromise belonging, place attachment, and security for existing residents. This study explores resistance to displacement through tenant advocacy and organizing in New York City. This research specifically focuses on the efforts of older, lower-income, African American women, who are most at risk for eviction and housing stability, and yet are at the center of advocacy efforts to preserve affordable, low-income housing. In three case studies, we interview key stakeholders invested in anti-displacement housing preservation, eviction resistance, and public housing organizing to highlight the often invisible work taking place from within socially vulnerable communities. Implications for policy and future directions for applied research are discussed.

